# Sublimation and Diffusion Kinetics of 2,4,6-Trinitrotoluene (TNT) Single Crystals by Atomic Force Microscopy (AFM)

**DOI:** 10.3390/molecules27175482

**Published:** 2022-08-26

**Authors:** Walid M. Hikal, Sanjoy K. Bhattacharia, Mark W. Vaughn, Brandon L. Weeks

**Affiliations:** 1 Department of Chemical Engineering, Texas Tech University, Lubbock, TX 79409, USA; 2 College of Engineering, West Texas A&M University, Canyon, TX 79016, USA

**Keywords:** 2,4,6-trinitrotoluene, activation energy, sublimation rates, explosives detection, diffusion coefficient, atomic force microscopy

## Abstract

In this article, we report the in-situ nanoscale experimental measurement of sublimation rates, activation energy of sublimation, and diffusion coefficients of 2,4,6-trinitrotoluene (TNT) single crystals in air using atomic force microscopy (AFM). The crystals were prepared by slow evaporation at 5 °C using acetone-dissolved TNT. The mass loss was calculated by monitoring the shrinkage of the surface area of layered islands formed on the surface of the TNT crystals due to sublimation upon isothermal heating at temperatures below the melting point. The results suggest the sublimation process occurs via two-dimensional detachment of TNT molecules from the non-prominent facets on the crystal surface which imitates the nucleation and crystal growth process. Sublimation rates are one order of magnitude smaller than previously reported values. However, the calculated activation energy (112.15 ± 3.2 kJ/mol) and temperature-dependent sublimation rates agree well with the reported values for TNT thin films and microcrystals determined by UV-vis absorbance spectroscopy and quartz crystal microscopy (QCM) (90–141 kJ/mol). The average diffusion coefficient is (4.35 × 10^–6^ m^2^/s) which is within the range of the reported theoretical values with an average of 5.59 × 10^–6^ m^2^/s, and about 25% less than that determined using thermogravimetric analysis for powder TNT.

## 1. Introduction

In the defense and security fields, detection of low volatile explosives and high energetic materials depends largely on accurate measurements of lifetime of these hazardous materials and their persistence. Meanwhile, measurement of the thermophysical parameters, such as sublimation rates and activation energy of sublimation, as well as the diffusion coefficient in air of an explosive in the nanoscale, could be used to evaluate its persistence on surfaces for trace detection as well as aging of these compounds upon long term storage.

Thermogravimetry analysis (TGA) is the most widely used technique in measuring sublimation rates of different materials. However, pressure build-up in TG often contributes to measurement inaccuracy and requires a flow of gas through both the balance chamber and the sample to prevent such error source [1,2,3]. In addition, the mass change detectable by TGA is in the order of a few nanograms, thereby limiting the usefulness to samples larger than a few milligrams, depending on the sensitivity of the balance. In addition, when used to measure sublimation rates of low volatile materials such as TNT, TGA will take days to weeks to collect sufficient data. Furthermore, TGA cannot be used for measuring sublimation from thin layers. This makes TGA an inappropriate technique to study thermophysical properties of large single crystals.

Quartz crystal microbalance (QCM) is a more sensitive tool for mass loss and has also been used in measuring sublimation rates in thin films [4,5]. QCM sensitivity to mass loss is also in the order of few nanograms with an uncertainty of about 15–20% when some materials’ mass loss could be in the order of attograms. Atomic force microscopy (AFM) has also been used to determine sublimation rates of thin nano-islands of explosives [6,7]. However, even when operated with special tips, the measurement of the volume of the nano-islands will lack accuracy due to shape irregularity and surface roughness.

UV-*vis* absorbance spectroscopy has also been used to measure the sublimation rates, activation energy of sublimation, and vapor pressure of low volatile materials and explosives such as TNT, 1,3,5-trinitroperhydro-1,3,5-triazine (RDX), and pentaerythritol tetranitrate (PETN), in the form of uniform continuous thin films [8], and results were compatible with those reported by using QCM technique [4,5].

The calculation of the surface area of a sample introduces errors in measuring the thermophysical properties of any sample in all discussed techniques with the advantage to UV-vis technique as there is no need to measure the surface area of the uniform thin film. This error is caused by the roughness of the surface of the sample. Surface roughness could vary from hundreds of microns to a few microns for single crystals and microcrystalline powders or non-uniform films, respectively. Cracks that could result in heating a material are also expected to introduce errors to the calculated surface area. In addition, the surface area of the sample is usually measured before performing the experiment and is assumed to be constant throughout the experiment which is not the real case as the sample sublimes/evaporates. Islands formed on crystal grown and used in this study have highly flat surfaces that allows for accurate surface area.

2,4,6-trinitrotoluene (TNT) is one of the most widely used explosive materials due to its high thermal stability, low cost, and ease of preparation, and it possesses low sensitivity to impact along with a high detonation power. TNT is a powerful secondary explosive used by both military (detonators, main charges boosters, and plastic explosives) and industry. To date, no sublimation or diffusion studies on single crystals of TNT have been made due to the fact that TNT crystallizes in the form of fragile small yellow crystalline needles.

The diffusion coefficient is an important factor required when modeling explosives alkaline hydrolysis treatment. The mass transfer rate-limiting step is most often controlled by diffusion. Therefore, to determine the rate of diffusion, it is necessary to measure diffusion coefficient. From a consideration of surface area available for sublimation it is the smaller particles which exhibit shorter lifetimes. A range of environmental parameters and particle properties influence particle sublimation including: the vapor pressure, particle geometry, temperature, airflow, relative humidity, condensation, inclusion chemicals, chemical adlayers, and solar load, [9,10,11].

TNT crystallizes in the form of small needles when formed by evaporation from a solvent at room temperature. Herein, we report the successful growth of millimeter size TNT crystals by slow evaporation from acetone at 5 °C. Atomic force microscopy is used to isothermally determine the sublimation rates and the activation energy of sublimation of TNT crystal, in air, below its melting/decomposition point by monitoring the shrinkage of a flat island on the surface of a single TNT crystal. The measured sublimation rates and the reported vapor pressures are then used to experimentally determine the diffusion coefficients of TNT single crystals in air.

## 2. Theory

Most high energy organic explosives exhibit melting point temperatures above possible ambient temperatures so their persistence as particles on a surface is largely controlled by sublimation. Sublimation can be described as two opposing vapor fluxes: solid–gas and gas–solid. The net sublimation or evaporation rate is the sum of the opposing vapor fluxes. The rate of mass loss (*dm/dt*) at temperature *T* in *Kelvin* is given by Arrhenius relation in the form [6,7]

(1) $$\frac{dm}{dt}=f\exp\left(-\frac{E_{a}}{RT}\right)\;\;$$

where f is the frequency factor, $E_{a}$ is the activation energy of sublimation, and R is the universal gas constant (8.314 JK^−1^mol^−1^). Considering a surface layer of area *A* and thickness *l*, and introducing the density $\left(\rho =\frac{m}{Al}\right)$, where *m* is the mass, into Equation (1), it takes the form:

(2) $$\frac{dA}{dt}=\frac{1}{\rho l}fexp\left(-\frac{E_{a}}{RT}\right)$$

where ρ is the density of the material, and $\frac{dA}{dt}$ is the areal sublimation rate per unit time. Hence, a plot of the $Ln\;\left[\frac{dA}{dt}\right]$ versus the inverse of the absolute temperature should give a straight line from which approximate values of the activation energy of sublimation (*E_a_*) and the frequency factor (*f*) can be calculated.

The determined sublimation rates along with the vapor pressures values reported in the literature at nano/microscale can be used to determine the diffusion coefficients of a material. Considering the quasi-stationary steady state diffusion process from the sample surface into the open space, the diffusive flow considerably depends on the sample morphology. Analytic solutions were derived for the cases of a uniform flat disk-shaped source. In this study, the diffusive flow from a disk-shaped sample is given by: [4,12]

(3) $$\frac{dm}{dt}=4r_{d}DC_{sat}M$$

where *dm/dt* is the diffusive flow from the source to open space in units of kg/s which is equal to rate of mass loss in Equation (1), *r_d_* is the radius of the disk, *D* is the diffusion coefficient, C*_sat_* represents the concentration of the saturated vapor, and M is the sample molecular mass. Assuming an ideal gas behavior (*P_sat_ = C_sat_ RT*) and a circular disk $\left(S=\pi r_{d}^{2}\right)$, the diffusion coefficient *D* can be determined by:

(4) $$D=\left(\frac{\pi r_{d}RT}{4MP_{sat}}\right)\left(\frac{dm}{dt}\right)$$

where *R* is the universal gas constant, *T* is the absolute temperature in Kelvin, and *P_sat_* is the saturation vapor pressure.

## 3. Results and Discussion

**Figure 1:**
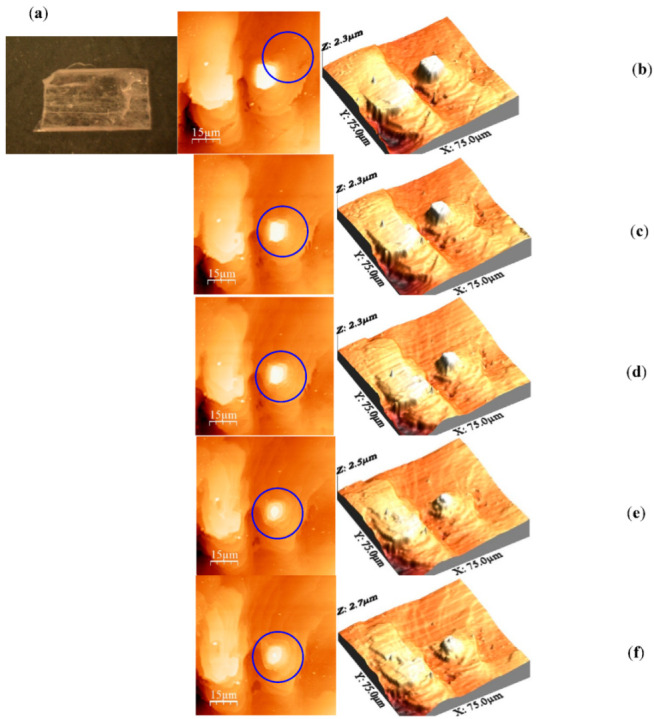
Optical image of TNT (**a**) and AFM final images (right) and their corresponding 3D images (left) of TNT crystal surface: (**b**) at room temperature, and heated at (**c**) 55 °C for 243 min, (**d**) 60 °C for 55 min, (**e**) 65 °C for 55 min, and (**f**) 70 °C for 30 min.

Figure 1a shows the optical image of about 6 mm × 4 mm × 3 mm TNT crystal. Figure 1b shows flat islands formed on the surface of a TNT crystal. Multiple flat islands are present on the crystal’s surfaces. As the figure shows, TNT crystals grow with two-dimensional nucleation and proceeded with layer-by-layer growth step. New flat layers were formed on the top of another layer without forming any spiral dislocation. The top of the island formed a semi rectangular smooth plane with a flat surface. The growth pattern suggested that TNT grows by incorporation of the laterally spread steps into an inland. Surface diffusion due to the likely high mobility of the TNT molecules might be the likely reason of the expansion of the layer. TNT crystal was heated isothermally in situ at different temperatures ranging from 55 °C to 70 °C for different periods suitable for sufficient data collection. As mentioned in the experimental section, images were recorded every 10 min at each temperature, to monitor the shrinkage rate of the surface area of the top layer.

Figure 1c–f represents the final images of the island after heating at the temperatures used in this study. The results clearly demonstrate the shrinkage of the island and gradual deviation from the semi rectangular morphology of the top layer. The images show the gradual evolution of new morphology when the material undergoes a kinetic process. This observation proves the basis of the assumption that desorption of solid to gas phase starts from kink sites when a molecular crystal is under thermal treatment.

**Figure 2:**
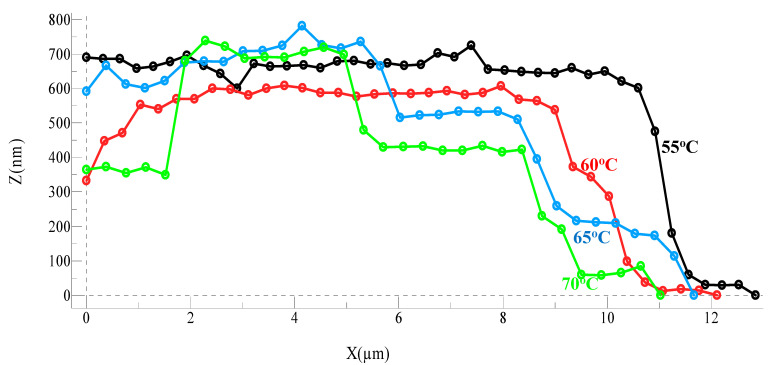
Profiles of island heights upon heating at different temperatures showing negligible decrease in height and the appearance of new crystal steps upon heating.

Figure 2 represents the profile of the island height (z-axis) versus the x-axis to examine the change in island. The island height is in the range of 600–700 nm, and clearly, there is no change in the island height upon heating the crystal for about six hours at different temperatures; however, increased surface roughness and a 2D shrinkage due to sublimation resulting in smaller steps of about 200–250 nm is observed. This indicates that the sublimation proceeds via the detachment of TNT molecules from the non-prominent facets on the crystal surface similar to the nucleation and crystal growth process for TNT and other explosives such as (PETN) [6].

**Figure 3:**
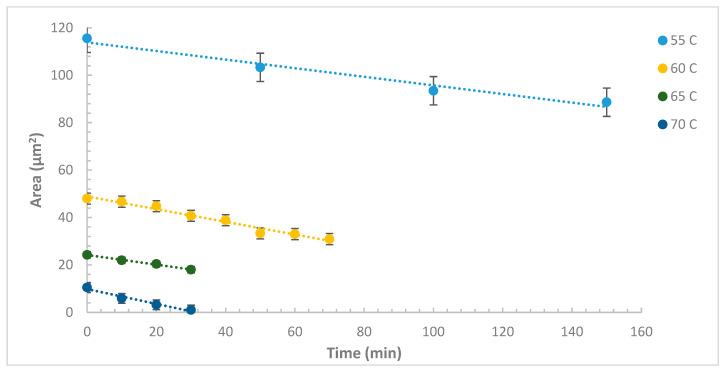
A plot of the surface area of the island vs. time at different temperatures from which the shrinkage rates are calculated.

Mass loss of TNT crystals $\rho =1.654\;g/{\mathrm{cm}}^{3}$, was calculated from the rate change in the island surface area upon heating. The sublimation rates calculated here are tabulated in Table 1. The sublimation rates are about one order of magnitude lower than those reported in the literature [4]. This could be due to the expected high crystalline nature of our samples compared to microcrystals and thin films which are expected to be partially crystalline. Plots of the surface area versus time was used to calculate the shrinkage rate at each temperature. As shown in Figure 3, the plots are linear with very good R values.

**Figure 4:**
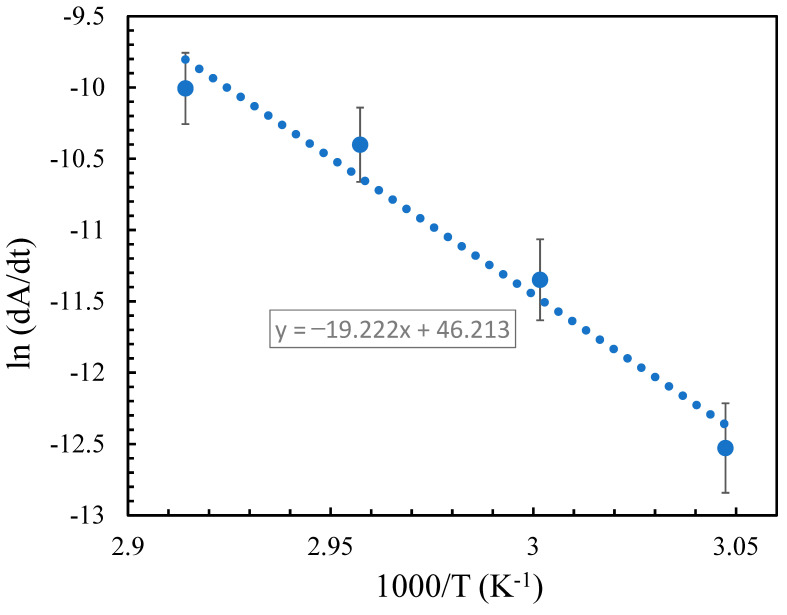
A plot of the logarithm of the shrinkage rates of TNT surface island *vs* the inverse of the absolute temperature, slope determines the activation energy of sublimation.

The log of the calculated shrinkage rates is plotted versus the inverse of the absolute temperature according to Arrhenius equation. As shown in Figure 4, the plot is linear, and the activation energy of sublimation is calculated to be 112.15 ± 3.2 kJ/mol. There is a large discrepancy in the sublimation activation energy value of TNT in the literature (90–141 kJ/mol) [4,12,13,14,15,16,17,18,19,20,21,22]. Our value is about 10% higher than the reported results using the most sensitive techniques used in measuring sublimation rate of TNT: QCM, and UV-vis absorbance spectroscopy, and Knudsen effusion methods giving activation energy of sublimation of 97 ± 7, 99.6 ± 5, and 103 kJ/mol, respectively [4,15,18]. An activation energy of sublimation of TNT micro-islands, that is particle size-dependent, has been reported (102–195.5 kJ/mol) [23]. Despite that the study was performed using AFM, it was carried out at temperatures below room temperature where coarsening of micro particles is expected to highly affect the calculated values. In addition, previous nano/microscale studies were performed on samples on substrates, where interfacial interactions are present and will introduce errors to the results. Thus, our results support the lower end values for TNT activation energy of sublimation that have been determined at nano/micro-scale where coarsening and interfacial interactions are not effective.

Diffusion coefficients are typically calculated using Equation (4). The diffusion coefficients of TNT were determined at the temperatures studied, and the values are summarized in Table 1.

**Table 1:** Comparison of diffusion coefficients of TNT single crystals and TNT powder and sublimation rates of TNT single crystals calculated here.

Diffusion Coefficient (D)			Sublimation Rates
	AFM	TGA [24]	AFM
T (°C)	*D* (m^2^/s)	*D* (m^2^/s)	$\left(dm/dt\right)$ ng/s
55	4.23 × 10^−6^	5.78 × 10^−6^	0.00015
60	4.31 × 10^−6^	5.79 × 10^−6^	0.00199
65	4.39 × 10^−6^	5.81 × 10^−6^	0.00231
70	4.48 × 10^−6^	5.82 × 10^−6^	0.00285

The reported theoretical diffusion coefficients of TNT diffusion coefficient show discrepancy of about 30% [24,25,26] with an average value of 5.59 × 10^–6^ m^2^/s. We have previously reported the average experimental value to be 5.8 × 10^–6^ m^2^/s using TGA for powder TNT, which is in excellent agreement with the average theoretical value [27]. The average diffusion coefficient calculated here is 4.35× 10^–6^ m^2^/s which is 25% less than the value calculated using TGA. The difference could be attributed to the great effect of the temperature on the mobility of the TNT surface molecules at the nano/micro-scale as the mobility increases at higher temperatures close to the melting point (82 °C for TNT). However, the value determined here agrees with those found in the literature [24,25,26]

## 4. Materials and Methods

TNT (Austin explosives) was purified by crystallization of TNT by means of ultrasonication in 2-isopropanol. Single crystal of TNT (6 × 3 × 2 mm) was grown in acetone by solvent evaporation in a glass vial at 5 °C in the dark in a refrigerator. TNT surface morphology was monitored with an atomic force microscope (AFM) operated in contact model with a silicon cantilever (Nanosensor pointprobes, Nominal spring constant 5.0 N/m) at 1.0 Hz scan rate. AFM was supplied by Veeco Instrument Inc., Santa Barbara, CA, USA). The TNT crystal was glued onto a metal piece which was heated using a High Temperature Heater (Bruker Corporation Inc., Billerica, MA, USA) directly connected to the sample holder of the AFM. AFM images were processed with *WSxM* 5.0 software. TNT sample is annealed with the built-in heater of the AFM at 55, 60, 65, and 70 °C. Processed AFM images were analyzed with ImageJ software [28].

## 5. Conclusions

Atomic force spectroscopy (AFM) was used to calculate the sublimation rates and activation energy of sublimation of TNT single crystals grown by evaporation from acetone solution at 5 °C. AFM was used to monitor the shrinkage of a single layer on the surface of a TNT surface island at different temperatures. The activation energy of sublimation is in excellent agreement with values determined at nanoscale with most accurate instrumentation. The sublimation rates calculated here are one order of magnitude smaller than the reported values. The sublimation rates and vapor pressure values reported in the literature were used to determine the diffusion coefficients of TNT. The determined diffusion coefficients values are about 40% smaller than the experimental values reported in the literature specially at temperatures closer to the melting point of TNT. However, values are within those values calculated theoretically.

## Data Availability

The data presented in this study are available on request from the corresponding author.

## Sample Availability

Samples of the compounds are not available from the authors.
